# Curcumin Attenuates Doxorubicin-Induced Cardiac Oxidative Stress and Increases Survival in Mice

**DOI:** 10.3390/pharmaceutics16081105

**Published:** 2024-08-22

**Authors:** Felipe S. Arruda, Fernanda D. Tomé, Anália C. Milhomem, Pablo I. R. Franco, Allisson B. Justino, Rodrigo R. Franco, Erica C. Campos, Foued S. Espindola, Danilo F. Soave, Mara Rubia N. Celes

**Affiliations:** 1 Department of Bioscience and Technology, Institute of Tropical Pathology and Public Health, Federal University of Goias, Goiania 74605-050, GO, Brazil; arrudaf@ymail.com (F.S.A.); fernandadiast@gmail.com (F.D.T.); analia.biomed.ufg@hotmail.com (A.C.M.); pablo_franco@discente.ufg.br (P.I.R.F.); 2 Laboratory of Biochemistry and Molecular Biology, Institute of Biotechnology, Federal University of Uberlandia, Uberlandia 38408-100, MG, Brazil; allissonbjustino@ufu.br (A.B.J.); rodrigo_franco@ufcat.edu.br (R.R.F.); foued@ufu.br (F.S.E.); 3 Department of Cardiovascular Physiotherapy, Faculty of Physical Education and Physiotherapy (FAEFI), Federal University of Uberlandia, Uberlandia 38408-100, MG, Brazil; ericacarol@gmail.com; 4 Morphofunctional Department, Faculty of Medicine of Goianesia (FAMEGO), University of Rio Verde—UniRV, Goianesia 76380-000, GO, Brazil; danilo.patologia.oral@gmail.com

**Keywords:** cardiotoxicity, curcumin, chemotherapy, antioxidants, oxidative stress, phytochemicals

## Abstract

Doxorubicin (DOX) is a potent chemotherapeutic agent used to treat multiple types of cancer, but its clinical application is limited by cardiotoxicity, mainly due to oxidative stress. Curcumin (CUR) is a natural polyphenolic compound with strong antioxidant properties, but its potential protective effects against DOX-induced cardiotoxicity need further investigation. This study aimed to evaluate CUR’s efficacy in mitigating DOX-induced oxidative stress in the hearts of BALB/c mice. Mice received a DOX dose of 9 mg/kg or 16 mg/kg; half of the mice received daily doses of 100 mg/kg CUR for 15 days. Survival analysis, histopathological examination, and oxidative stress markers were assessed to determine the cardioprotective effects of CUR. Results showed that CUR significantly reduced oxidative damage and improved survival rates, particularly at the lower DOX dose (9 mg/kg). Mice treated with DOX-9 mg/kg plus CUR showed improved health conditions and reduced levels of reactive oxygen species (ROS), lipid peroxidation, sulfhydryl production, and protein carbonylation. Histopathological analysis confirmed reduced cardiac tissue damage. In conclusion, CUR combined with a lower dose of DOX effectively reduces oxidative stress and cardiac injury, enhancing survival in BALB/c mice. These findings suggest that CUR is a promising adjunct therapy to mitigate DOX-induced cardiotoxicity, potentially improving the DOX therapeutic index in cancer treatment.

## 1. Introduction

Doxorubicin (DOX) is a highly effective chemotherapeutic agent belonging to the anthracycline antibiotics class, commonly used to treat a variety of cancers, including sarcomas, carcinomas, and hematological malignancies [1,2,3]. Despite its high efficacy, since the clinical discovery of DOX, several toxic effects associated with its treatment have been recorded, notably cardiotoxicity, which can manifest either acutely or chronically following treatment [4]. The heart’s susceptibility to DOX-induced damage is primarily due to the drug’s mechanisms, which include mitochondrial injury, alterations in sarcoplasmic reticulum and myofibrils, loss of interstitial collagen, disruptions in Ca^2+^ channels and excitation-contraction coupling, modifications in iron metabolism, and the promotion of oxidative stress [5,6,7,8]. 

DOX interacts with the electron transport chain, increasing the production of reactive oxygen and nitrogen species (ROS/RNS), leading to oxidative damage of mitochondrial biomolecules and increased membrane permeability. This process directly interferes with its biological functions and contributes to the accumulation of cellular injuries, ultimately compromising cell viability [9,10]. Although DOX-induced toxicity affects multiple organs, cardiac toxicity remains a significant challenge due to its superior efficacy in eradicating neoplastic cells compared to other pharmacological strategies [11,12]. The DOX cardiotoxicity is still a challenging topic due to its important anti-neoplastic therapeutic action. Therefore, it is necessary to develop new pharmacological agents that can mitigate the toxic effects of DOX without compromising its therapeutic efficacy against cancer [13].

In this context, phytochemicals have accumulated substantial attention for their potential to provide disease protection and prevention without adversely affecting clinical DOX activity or producing long-term risks [14,15]. Among these natural compounds, curcumin (CUR), derived from *Curcuma longa* L., has shown considerable promise due to its anti-inflammatory and antioxidant properties [16,17]. Additionally, CUR acts as a natural antioxidant, protecting cellular components from oxidative damage and reducing free radicals [18]. Therefore, this study aims to evaluate the cardioprotective effects of CUR on the development of cardiac lesions, biochemical parameters, and oxidative stress in DOX-treated mice.

## 2. Materials and Methods

### 2.1. Experimental Animals

**Figure 1:**
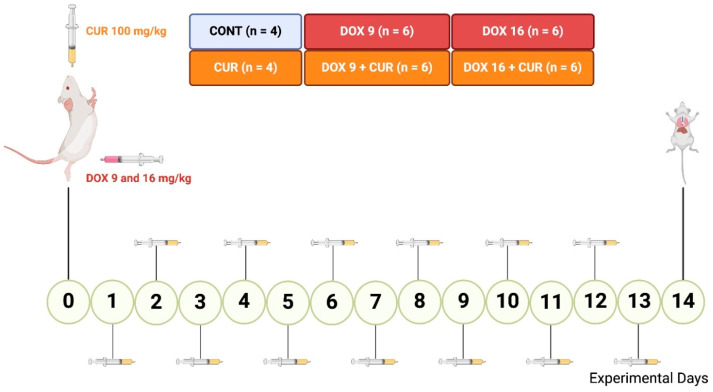
Experimental Design. Schematic representation of the experimental protocol including doxorubicin (DOX) injections at doses of 9 mg/kg and 16 mg/kg (i.p.), daily curcumin (CUR) treatment at 100 mg/kg (g.v.), and the timeline leading to the euthanasia of the animals at the end of the 15-day experimental period. Created with BioRender.com. https://app.biorender.com/illustrations/664252097fba1fad22dd9b61?slideId=fe0de6e5-063a-4949-a853-8676af1cce42 (accessed on 13 May 2024).

This study was conducted at the Institute of Tropical Pathology and Public Health (IPSTP) at the Federal University of Goiás (IPTSP/UFG), Goiânia, Brazil following approval from the Ethical Committee on the Use of Animals (CEUA, protocol 050/2016). Thirty-two adult male BALB/c mice weighing 30–35 g were used. The mice were housed under controlled environmental conditions with a mean temperature of 24 °C ± 2 °C and a 12-h light/dark cycle. They were provided with a solid laboratory diet and water ad libitum. The mice were randomly divided into six groups with the following treatments. (1) CT: mice injected intraperitoneally (i.p.) with a single dose of sterile saline (100 µL i.p.); (2) CUR: mice received a daily gavage (gv.) of 100 mg/kg curcumin (CUR) suspended in distilled water; (3) DOX-9: a single dose of DOX (9 mg/kg i.p.); (4) DOX-9+CUR: a single dose of DOX (9 mg/kg i.p.) and daily gavage of 100 mg/kg CUR suspended in distilled water; (5) DOX-16: single dose of DOX (16 mg/kg i.p.); and (6) DOX-16+CUR: a single dose of DOX (16 mg/kg i.p.) and daily gavage of 100 mg/kg CUR suspended in distilled water. The treatment protocol was conducted for 15 days, with CUR administration starting on the first day, two hours after the DOX injection, and continuing daily. Throughout the study, the animals were monitored for survival and general health, including weight changes and signs of distress. The experimental schedule is shown in Figure 1. 

### 2.2. DOX and CUR Treatment

Doxorubicin hydrochloride solution (Merck, Darmstadt, Germany) was administered intraperitoneally (i.p.) at concentrations of 9 and 16 mg/kg on experimental day 0 (zero). Control group animals (CT) received a single injection of sterile saline solution (100 μL i.p.) following the same protocol as the DOX-treated groups. 

For the CUR treatment, curcumin (CUR) was provided by the University Pharmacy of UFG, Goiânia, Brazil (Lot 057939, Dry Extract > 96%, manufactured in 09/2015). CUR was administered by gavage (g.v.) at a dose of 100 mg/kg, suspended in 100 μL of distilled water, every morning. Specifically, on the first day of the experiment, curcumin was administered by gavage 2 h after the DOX injection and continuing at 24-h intervals for each subsequent dose. The same scheme was applied to the animals in the CUR. This treatment was given daily for 15 consecutive days. At the end of the 15-day treatment period, the mice were euthanized, and their hearts were harvested for histopathological and biochemical analyses to assess the extent of cardiac lesions and oxidative stress markers.

### 2.3. Histopathological Analysis of Cardiac Tissue

Following euthanasia, the hearts of the mice were collected and immediately fixed. The fixed tissues were then processed and embedded in Paraplast (Leica Paraplast) to preserve the tissue morphology. Histological sections were prepared, and approximately 5 μm thick sections were mounted on glass slides and stained with hematoxylin and eosin (HE) for morphological evaluation [19]. The stained sections were analyzed using a light microscope (Leica DMC 2900) connected to a computer equipped with LAS software (version 4.4.0 [Build 454], Leica Microsystems CMS GmbH, Mannheim, Germany, 2003–2013). This setup enabled detailed histopathological examination and documentation of the cardiac tissue morphology. The analysis focused on identifying and characterizing lesions, assessing tissue integrity, and detecting any pathological changes induced by the treatments.

The morphological evaluation of cardiac tissue stained with hematoxylin-eosin (HE) was performed using a scoring system to classify alterations as follows: discrete (+), moderate (++), or accentuated (+++). Based on established criteria, the assessment considered myocytolysis, nuclear alterations (pyknosis, karyolysis, and karyorrhexis), membrane and cytoplasmic changes, inflammatory infiltration, and fibroblasts [19,20,21]. 

### 2.4. Biochemical Evaluation of Oxidative Stress

For oxidative stress markers, cardiac tissue samples were homogenized in phosphate buffer. The homogenates were centrifuged, and the total protein concentration in the supernatant of the samples was measured by the Bradford method [22].

ROS: Samples were incubated with dichlorodihydrofluorescein diacetate and Tris-HCl buffer. Afterwards, the fluorescence reading was performed at 474 nmex and 530 nmem [23].

Sulfhydryls: Thiol groups were evaluated using dithionitrobenzoic acid (DTNB) diluted in potassium phosphate buffer. The homogenates were incubated with phosphate buffer and DTNB solution. The presence of sulfhydryl groups was detected at 412 nm [24].

Lipid peroxidation: Lipid peroxidation was determined according to thiobarbituric acid reactive substances (TBARS) by the reaction method between malondialdehyde (MDA) present in heart samples and thiobarbituric acid. Lipid peroxidation was determined using an analytical curve constructed with MDA [25]. 

Protein carbonylation: Carbonyls in proteins were identified by 2,4-dinitrophenyl-hydrazine (DNPH), precipitated with trichloroacetic acid (TCA), washed with ethanol-ethyl acetate, and dissolved in L-1 guanidine hydrochloride. The absorbance values were recorded at 370 nm (Molecular Devices, Menlo Park, CA, USA), and protein carbonylation was determined according to the difference between the spectrum of the sample treated with DNPH in HCl and that of the sample treated only with HCl [25].

Catalase activity: The evaluation of CAT activity was based on hydrogen peroxide decomposition by CAT present in heart samples. The homogenates were incubated with Triton X-100 and potassium phosphate buffer containing hydrogen peroxide. The decomposition of hydrogen peroxide was monitored at 240 nm for 10 min (PerkinElmer LS 55, Waltham, MA, USA) [25].

Total antioxidant capacity: The total antioxidant capacity of cardiac tissue was assessed by the ferric reducing antioxidant power (FRAP). The heart homogenate was incubated with the reagent, and the absorbance was measured at 593 nm (Molecular Devices, Menlo Park, CA, USA) [24].

Superoxide dismutase activity: SOD activity was evaluated by the inhibition of pyrogallol autoxidation by SOD present in the heart. Cardiac homogenates were mixed with Tris-Buffer HCl and EDTA. The kinetic assay was monitored for 10 min at 420 nm (Molecular Devices) using an analytical curve constructed with SOD as a standard [26].

Reduced glutathione: Homogenated proteins were precipitated with metaphosphoric acid. The supernatant was mixed with sodium phosphate buffer containing EDTA, and fluorescence was measured at 350 nm and 420 nm (PerkinElmer LS 55, Waltham, MA, USA) [26]. 

### 2.5. Statistical Analysis

Statistical analyses were conducted using GraphPad Prism 8 (GraphPad Software Inc., San Diego, CA, USA). The D’Agostino–Pearson test was initially applied to assess the normality distribution of the data. For multiple group comparisons, analysis of variance (ANOVA) followed by Tukey’s post hoc test was used to determine significant differences between groups. Survival analysis was performed using Kaplan–Meier survival curves, and the log-rank test was employed to evaluate significant differences between survival distributions. All statistical tests were two-tailed, and a significance level of 5% (*p* < 0.05) was considered statistically significant. Results are presented as the mean ± standard deviation (SD).

## 3. Results

### 3.1. Curcumin Reduces DOX-Induced Mortality in Mice

**Figure 2:**
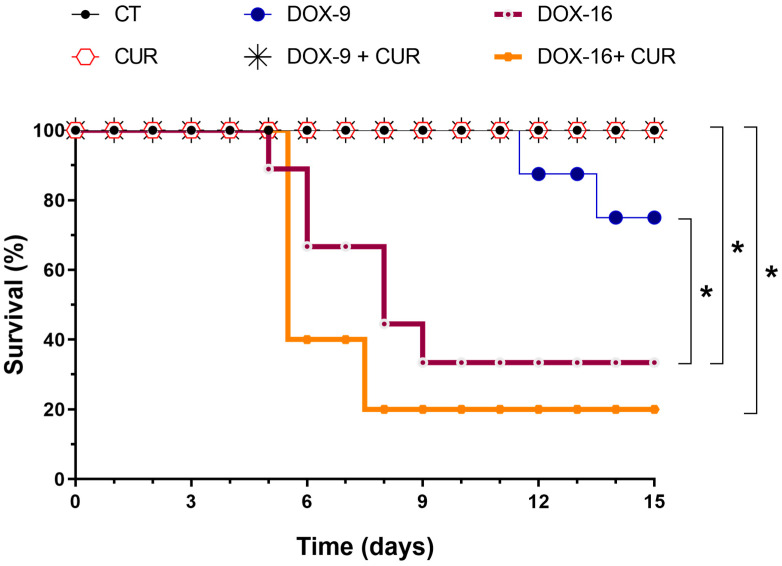
Survival rates of experimental groups. Control group (CT), curcumin-treated group (CUR), 9 mg/kg doxorubicin (DOX-9), 9 mg/kg doxorubicin daily treated with 100 mg/kg curcumin (DOX-9+CUR), 16 mg/kg doxorubicin (DOX-16), and 16 mg/kg doxorubicin daily treated with 100 mg/kg curcumin (DOX-16+CUR). Survival analysis was performed using Kaplan–Meier curves, and statistical significance was assessed with the log-rank test. Significant differences were observed between the following comparisons: CT vs. DOX-16 (*p* < 0.05), DOX-9 vs. DOX-16 (*p* < 0.05), DOX-9+CUR vs. DOX-16+CUR (*p* < 0.05), and CUR vs. DOX-16+CUR (*p* < 0.05). * Statistical difference between the compared groups.

Figure 2 shows the survival rates of mice across the different experimental groups. At the end of the experimental period, the survival rate in the DOX-9 group was 75%, significantly lower than the 100% survival observed in both the CT and CUR groups. However, the DOX-9+CUR group demonstrated a survival rate comparable to the CT and CUR groups, indicating a protective effect of CUR at this lower DOX dose. In contrast, treatment with the higher concentration of DOX (DOX-16) resulted in a substantially higher mortality rate, with only 35% of the mice surviving until the end of the study period. While adding CUR to the high-dose DOX treatment (DOX-16+CUR) did not achieve survival rates as high as those in the CT and CUR groups, it was evident that CUR does not improve survival compared to the DOX-16 group alone. A comparison between the DOX-9+CUR and DOX-16+CUR groups revealed that CUR was more effective at reducing mortality in the lower DOX dose group. These findings suggest that CUR cardioprotective effects are more pronounced at lower levels of DOX-induced cardiotoxicity.

### 3.2. Protective Effects of CUR on DOX-Induced Cardiac Tissue Damage

**Figure 3:**
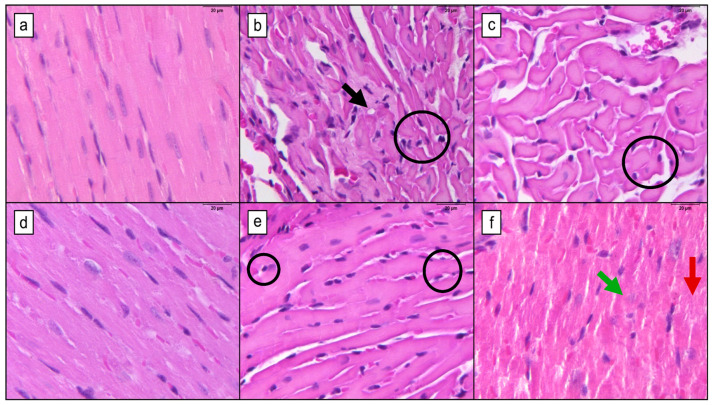
Histopathological findings in mice treated with or without doxorubicin (DOX) and curcumin (CUR). (**a**) CT group; (**b**) DOX-9 group; (**c**) DOX-16 group; (**d**) CUR group; (**e**) DOX-9+CUR group; and (**f**) DOX-16+CUR group. Black arrows indicate cytoplasmic vacuolization, circles highlight perinuclear halos, green arrows denote karyolysis, and red arrows point to myocytolysis. Calibration bar = 20 µm; final magnification = 400×.

DOX treatment resulted in a noticeable increase in interstitial space and a range of structural alterations in cardiac tissue (Figure 3). In the DOX-9 group, histological examination revealed focal areas of myocytolysis and hyaline degeneration, characterized by cells with a homogeneous hyaline appearance indicative of protein microfilament disintegration. Marked and diffuse nuclear, membrane, and cytoplasmic alterations were also observed. The combination of DOX-9 and CUR (DOX-9+CUR) significantly attenuated these lesions, which appeared moderate and dispersed rather than diffuse. In contrast, the DOX-16 group exhibited more extensive myocytolysis, hyaline degeneration, and diffuse nuclear, membrane, and cytoplasmic alterations, alongside a pronounced increase in interstitial space, highlighting the severity of the cardiac damage at this higher DOX dose. Treatment with CUR in the DOX-16+CUR group did not substantially mitigate the development of lesions or modifier the morphological pattern of observed alterations compared to the DOX-16 group. These findings suggest that the protective effects of CUR are limited at higher DOX concentrations.

### 3.3. Evaluation of Oxidative Stress

**Figure 4:**
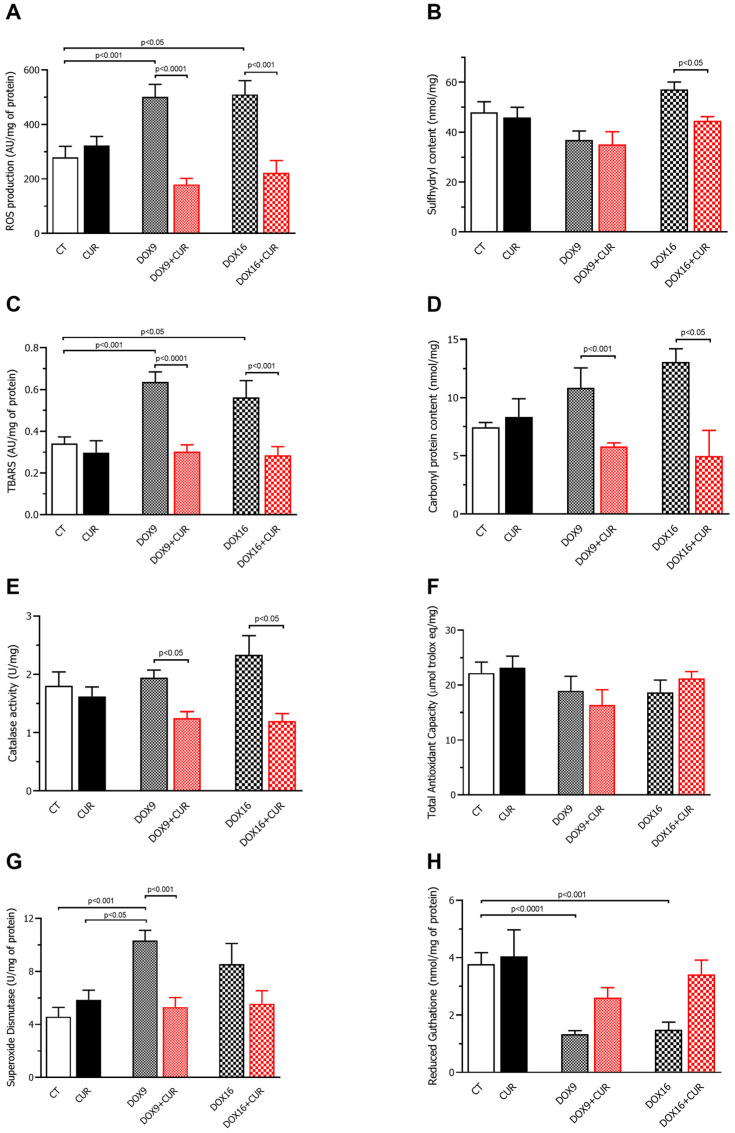
Oxidative stress in cardiac tissue. Measurements include reactive oxygen species (ROS) (**A**), sulfhydryl groups (**B**), thiobarbituric acid reactive substances (TBARS) (**C**), carbonyl proteins (**D**), catalase activity (**E**), total antioxidant capacity (**F**), superoxide dismutase (SOD) activity (**G**), and reduced glutathione (GSH) (**H**). The experimental groups are as follows: control (CT), doxorubicin 9 mg/kg (DOX-9), doxorubicin 16 mg/kg (DOX-16), and groups treated daily with 100 mg/kg curcumin (CUR), DOX-9+CUR, and DOX-16+CUR. Statistical analysis was performed using ANOVA and Tukey’s test for multiple comparisons, with significance set at *p* < 0.05. Data are expressed as mean ± SD.

Reactive oxygen species (ROS): ROS levels were significantly elevated in both DOX-9 and DOX-16 groups compared to controls, indicating that DOX increases ROS production irrespective of the dose. However, CUR treatment considerably reduced ROS levels in both DOX-9+CUR and DOX-16+CUR groups, suggesting CUR’s efficacy in mitigating oxidative stress induced by DOX (Figure 4A).

Sulfhydryls: The DOX-9 treatment did not result in a statistically significant change in sulfhydryl group levels. Conversely, the DOX-16+CUR group showed a significative reduction in sulfhydryl groups upon CUR treatment, indicating CUR’s potential role in protecting sulfhydryl groups at higher DOX concentrations (Figure 4B).

Lipid peroxidation: Lipid peroxidation, measured via thiobarbituric acid reactive substances (TBARS), was increased in both DOX-9 and DOX-16 groups. CUR treatment restored TBARS levels to those observed in the CUR groups (DOX-9+CUR and DOX-16+CUR), highlighting CUR’s ability to counteract DOX-induced lipid peroxidation (Figure 4C).

Carbonyl proteins: CUR treatment reduced the levels of carbonylated proteins in both DOX-9 and DOX-16 groups. As carbonylated proteins are markers of oxidative damage, this result underscores CUR’s protective effect against DOX-induced oxidative damage (Figure 4D).

Catalase activity: Catalase activity did not increase in groups treated with DOX alone. However, CUR treatment significantly reduced catalase enzyme activity in both DOX-9+CUR and DOX-16+CUR groups, suggesting an interaction between CUR and catalase activity (Figure 4E).

Total antioxidant capacity (FRAP): No significant differences in total antioxidant capacity were observed among the groups, indicating that this measure may not fully capture CUR’s antioxidant effects (Figure 4F).

Superoxide dismutase (SOD): SOD levels were significantly increased in the DOX-9 group compared to the CT and CUR groups. CUR treatment in the DOX-9 groups (DOX-9+CUR) resulted in a significant reduction in SOD activity compared to the DOX-9 group, suggesting that CUR modulates SOD activity in response to DOX-induced oxidative stress (Figure 4G).

Glutathione (GSH): DOX treatment significantly reduced GSH levels. Although CUR treatment altered the GSH levels, it did not significantly inactivate free radicals, indicating a complex interaction in the antioxidant response (Figure 4H).

## 4. Discussion

The present study investigated the protective effects of curcumin against doxorubicin-induced cardiotoxicity. Curcumin treatment significantly improved survival rates in the DOX-9+CUR group compared to the DOX-9 group alone. CUR’s efficacy was evident in its ability to enhance superoxide dismutase (SOD) levels while reducing reactive oxygen species (ROS) in cardiac tissue.

Oxidative stress is a critical factor in the progression of DOX-induced myocardial dysfunction. The semiquinone form of DOX is a short-lived metabolite that interacts with molecular oxygen, generating ROS such as superoxide radicals, hydroxyl radicals, and hydrogen peroxide. These ROS induce oxidative damage on critical cellular components, including mitochondrial and cell membranes, culminating in the death of cardiomyocytes. DOX exacerbates oxidative stress by diminishing the activity of antioxidant enzymes such as SOD and catalase (CAT), thereby increasing the susceptibility of cardiomyocytes to oxidative damage more than other tissues [27,28]. 

DOX’s primary mechanism of action involves intercalation into double-stranded DNA, disrupting the cell cycle through interactions with topoisomerase II isoforms [29]. DOX paralyzes the cell cycle of neoplastic cells and several types of cells that multiply rapidly, such as gastrointestinal cells, capillaries, and bone marrow blood precursors, promoting toxicity to various tissues and cells [30]. During this process, the ROS generated causes marked changes in metabolism and cellular damage, leading to toxicity and cell death, if not reversible [31]. Thus, the resultant ROS accumulation and cell damage underlines the necessity for interventions that mitigate oxidative stress. Among the most recent alternatives proposed, curcumin, known for its potent anti-inflammatory and antioxidant properties, has garnered attention for its clinical applications [32]. Curcumin, the primary curcuminoid of *Curcuma longa* L. rhizomes, is a powerful fat-soluble antioxidant, capable of acting as an efficient donor of hydrogen atoms in aqueous solutions [33,34,35,36]. 

Katamura et al. (2014) demonstrated that curcumin improved survival rates in mice treated with DOX (20 mg/kg, single dose i.p.), indicating the dose-dependent protective effects of CUR. In the first week, 48% of the mice died; the other 52% died by the end of the fourth week of the study [37]. According to our analysis of survival rates in different treatment groups, concomitant administration of DOX-9 and CUR significantly improved survival compared with treatment with DOX-9 alone. However, groups treated with a higher dose of DOX (16 mg/kg) exhibited high mortality rates, and daily administration of CUR did not improve survival. These findings highlight the dose-dependent mortality associated with doxorubicin and indicate that curcumin is particularly effective at lower doxorubicin concentrations.

Considering the generation of ROS through the administration of DOX, the antioxidant capacity and harmful oxidative elements in the hearts of mice were analyzed. In our study, CUR treatment reduced ROS production in DOX-treated mouse heart tissue. Additionally, HE et al. (2018) reported the efficacy of CUR in mitigating DOX cardiotoxicity through the prevention of mitochondrial dysfunction, highlighting the role of mitochondrial membrane stabilizing proteins such as Bcl-2 [38]. Additionally, we found a reduction in lipid peroxidation through treatment with CUR. Lipid peroxidation analysis, using sulfhydryl and thiobarbituric acid reactive substances (TBARS) [39], indicated that CUR effectively diminished the oxidative degradation of lipids, preserving cell membrane integrity and selective permeability [40], maintaining the hydroelectrolytic balance between the cell and the extracellular matrix [40]. According to our findings, CUR reduced the amount of carbonylated proteins, and standard oxidative stress markers [38,41], suggesting its protective role against protein oxidation.

The chemical constitution of curcumin is based on phenolic compounds, which scavenge free radicals, preventing lipid peroxidation and, consequently, protecting cells, organelles, and macromolecules, including DNA, from oxidative damage [42]. Assays for FRAP, catalase activity, SOD, and GSH, performed to verify the antioxidant capacity, revealed that, in our experimental model, the analysis of FRAP and GSH after CUR treatment was not different, even though it has been reported that CUR has the potential to increase superoxide dismutase (SOD), catalase (CAT), glutathione peroxidase (GPx) and glutathione S-transferase (GST) enzyme activities [43]. Catalase breaks down harmful hydrogen peroxide into oxygen and water, SOD catalyzes the conversion of superoxide radical (O_2_^−^) into hydrogen peroxide (H_2_O_2_), preventing harmful oxidative processes, and GSH is an intracellular antioxidant that reacts with an unpaired electron from a free radical using NADPH as a substrate [44,45]. In contrast, we observed that CUR reduced catalase activity and SOD levels (DOX-9+CUR), suggesting the CUR efficacy in eliminating reactive species that induce oxidative stress. Furthermore, decreased injury or preservation of cardiac myocyte structure can be attributed to the potential antioxidant effect of curcumin. However, more studies are needed to elucidate the exact mechanisms by which curcumin influences oxidative stress and its impact on antioxidant enzymes such as catalase and SOD. This could include investigating whether the observed decrease in enzyme activity results from increased antioxidant efficiency, enzyme inhibition, or depletion of the antioxidant defense system.

Although doxorubicin (DOX) is commonly administered in cumulative doses in many studies, our experimental design focused on using two different concentrations in a single dose. This approach was chosen to effectively induce acute and subclinical toxicity within the timeline of our study. By doing so, we explored the immediate and delayed cardiotoxic effects of DOX, which is particularly relevant for understanding the onset of cardiotoxicity induced by this drug. This methodology is supported by similar findings in the literature, such as the study by Zhang et al. (2022), which also explored DOX-induced delayed-onset cardiotoxicity in mice [46]. By employing different dosage levels of DOX, our approach provides a more comprehensive understanding of its cardiotoxic effects, offering insights into both acute and subclinical toxicity. This is particularly relevant for clinical trials, where curcumin could be investigated as a potential cardioprotective agent against DOX-induced cardiotoxicity. Understanding the dose-dependent effects of DOX and CUR’s role in mitigating these effects is crucial for developing effective therapeutic strategies.

Regarding bioavailability, it is well documented that CUR faces significant challenges due to its low water solubility, leading to reduced bioavailability. Several strategies have been proposed to increase the bioavailability of CUR, including solubilization in oils, lipid-based formulations, and nanosystems [47]. Promising approaches, given the need to improve the therapeutic efficacy of CUR, increase its absorption and systemic availability [47]. In our study, CUR was suspended in distilled water, which represents a limitation given its poor solubility. Despite this limitation, CUR was still able to exert a measurable antioxidant effect, as evidenced by our analysis of oxidative stress biomarkers. However, the water as a suspension medium may have restricted the full potential of CUR’s therapeutic effects. Moreover, our study did not include functional cardiac examinations such as echocardiography or electrocardiography, which would have provided critical insights into the functional outcomes of CUR treatment on cardiac performance. Incorporating these evaluations in future studies could help clarify the relationship between CUR antioxidant effects and the prevention of pathophysiological changes observed in cardiac tissue, thus offering a more comprehensive understanding of its cardioprotective potential.

## 5. Conclusions

In conclusion, our findings demonstrate that curcumin treatment significantly improved survival rates in mice exposed to lower concentrations of DOX, presented a potent antioxidant effect, mitigating the harmful oxidative processes induced by doxorubicin, and effectively neutralized or inactivated reactive species responsible for cardiac injury, thus highlighting its potential as a cardioprotective agent in chemotherapy protocols involving DOX.

## Data Availability

The original contributions presented in the study are included in the article, further inquiries can be directed to the corresponding author.
